# Activation of ER stress signalling increases mortality after a major trauma

**DOI:** 10.1111/jcmm.15548

**Published:** 2020-08-18

**Authors:** Abdikarim Abdullahi, Dalia Barayan, Roohi Vinaik, Li Diao, Nancy Yu, Marc G. Jeschke

**Affiliations:** ^1^ Faculty of Medicine University of Toronto Toronto ON Canada; ^2^ Biological Sciences Sunnybrook Research Institute Toronto ON Canada; ^3^ Ross Tilley Burn Centre Sunnybrook Hospital Toronto ON Canada; ^4^ Department of Surgery Division of Plastic Surgery and Department of Immunology University of Toronto Toronto ON Canada

**Keywords:** burns, ER stress, hypermetabolism, liver, trauma, unfolded protein response

## Abstract

The endoplasmic reticulum (ER) adapts to stress by activating a signalling cascade known as the ER stress response. While ER stress signalling is a central component of the cellular defence against environmental insult, persistent activation is thought to contribute to the progression of various metabolic complications via loss of protein function and cell death. Despite its importance however, whether and how ER stress impacts morbidity and mortality in conditions of hypermetabolism remain unclear. In this study, we discovered that chronic ER stress response plays a role in mediating adverse outcomes that occur after major trauma. Using a murine model of thermal injury, we show that induction of ER stress with Tunicamycin not only increased mortality but also resulted in hepatic damage and hepatic steatosis. Importantly, post‐burn treatment with chaperone ER stress inhibitors attenuated hepatic ER stress and improved organ function following injury. Our study identifies ER stress as a potential hub of the signalling network affecting multiple aspects of metabolism after major trauma and as a novel potential molecular target to improve the clinical outcomes of severely burned patients.

## 1 INTRODUCTION

Metabolic ‘re‐wiring’ is a hallmark of a number conditions such as cancer, heart disease, burns that enables cells to survive, thrive, and to meet the heightened energy demands following injury.^1^, ^2^, ^3^, ^4^ Consequently, numerous signalling pathways are reprogrammed to direct enhanced nutrient acquisition via the mobilization of lipids and proteins. Within the hierarchy of pathways altered during the hypermetabolic state is the endoplasmic reticulum (ER), a key site for maintaining the function and integrity of cellular homeostasis.^5^ Due to its central role as a protein quality control apparatus, the ER is responsible for mediating proper protein folding, maturation and secretion.^5^ However, various pathological stimuli that increase secretory protein production or decrease folding capacity in the ER lumen can lead to the accumulation of unfolded or misfolded proteins. The ER senses and responds to the perturbations within the compartment by initiating a signalling cascade known as the ER stress response. In an effort to re‐establish equilibrium, the ER then triggers a defence mechanism referred to as the unfolded protein response (UPR).^5^ This results in the inhibition of secretory protein translation and transcriptional up‐regulation of ER chaperone proteins to help restore proper protein folding and function.^6^, ^7^, ^8^, ^9^ If the UPR fails to resolve ER stress and protein homeostasis, alternative apoptotic cell death pathways are activated.^10^

While primarily adaptive in nature, prolonged ER stress signalling is a major contributor to several disease states including type II diabetes and metabolic syndrome.^11^, ^12^ Although persistant hypermetabolism has also been associated with significant morbidity and mortality in a number of conditions, it is currently unclear whether and how these symptoms are interconnected. Interestingly, the ER stress response was recently proposed to be one of the critical signalling pathways mediating the catastrophic consequences of the unrestrained hypermetabolic response such as inflammation, insulin resistance (IR) and multi‐organ failure.^13^, ^14^, ^15^ Indeed, hypermetabolic patients, including those with cancer and trauma, often suffer from metabolic dysfunction in the liver, an organ enriched in both smooth and rough ER.^16^, ^17^ In fact, acute liver failure as a result of hypermetabolic reprogramming is associated with a rapid and aggressive course of clinical deterioration and a very poor prognosis in burns.^18^, ^19^ As the myriad of metabolic functions performed by the liver is compartmentalized in the ER, it is not known whether and how ER homeostasis and UPR signalling impact hepatic function and apoptosis during periods of hypermetabolism.

The current study addressed the question whether augmented ER stress signalling contributes to poor survival outcomes and organ failure in hypermetabolic conditions. Therefore, we investigated the role of ER stress signalling in a well‐established mouse model of trauma‐induced hypermetabolism. Our findings indicate that acute activation of the ER stress response after severe burn injury directly affects morbidity and mortality. Post‐burn ER stress signalling resulted in hepatic damage and hepatic steatosis, whereas treatment with chemical chaperones that inhibit the ER stress response significantly improved organ function following injury. Our data validate the contribution of ER stress to the adverse outcomes after major trauma, and alleviation of this response may represent an attractive means to treat hypermetabolism and its related metabolic disorders.

## 2 MATERIALS AND METHODS

### 2.1 Animals and diets

Male C57BL/6 (7 weeks old) were obtained from Jackson Laboratories (Maine, USA) and were housed at thermoneutral temperature (28°C) for one week prior to studies to acclimatize and cared in accordance with the Guide for the Care and Use of Laboratory Animals. All procedures performed in this study were approved by the Sunnybrook Research Institute Animal Care Committee under the animal use protocol (AUP) 467; (Toronto, Ontario, Canada).

### 2.2 Mouse burn injury model

Mice were anaesthetized using ketamine (0.15 mg/g) and xylazine (0.01 mg/g). The dorsum of the trunk was shaved and 1.5 ml Ringer's Lactate was injected subcutaneously along the spine and 0.5 ml Ringer's Lactate was injected intraperitoneally. Mice were placed in a mould that exposed 30% total body surface area of the shaved dorsum. A full thickness cutaneous scald burn was administered by lowering the mould into a 98°C water bath for 10 s. Mice in the sham group were shaved, anaesthetized and received Ringer's Lactate.

### 2.3 Induction of ER stress by tunicamycin

Tunicamycin from Streptomyces sp (Sigma) was dissolved in dimethyl sulphoxide (DMSO) and diluted in sterile 150 mM dextrose to obtain a tunicamycin concentration of 10 µg/µl. Mice (20‐25 g) were randomized into 3 groups, in which they either received a burn injury, or injected intraperitoneally with one dose of tunicamycin solution (1 µg/g body mass) either 24 hours pre‐burn or 24 hours post‐burn and assessed for survival. As controls, mice were injected intraperitoneally with control buffer (150 mM dextrose containing 1% DMSO).

### 2.4 Western blotting

Liver tissues were lysed in RIPA buffer (50 mM Tris‐HCl pH 7.5, 150 mM NaCl, 1% Igepal, 0.5% sodium deoxycholate, 0.1% SDS, 1 mM NaF and protease inhibitors) using tissue beater. Total protein was separated on SDS‐PAGE gels, transferred to nitrocellulose membrane and incubated with primary antibodies directed against p‐eif2a, CHOP, Bip (Cell Signaling), Cleaved Caspase‐3 (Cell Signaling), IRE‐ 1(Cell Signaling), Perk (Cell Signaling), ATF‐6 (Cell Signaling) and GAPDH (Cell Signaling). Proteins were visualized by enhanced chemiluminescence.

### 2.5 Hepatocyte apoptosis and damage assays

Serum alanine aminotransferase (ALT) and Aspartate aminotransferase (AST) were measured using assay kits (abcam). Formalin‐fixed, paraffin‐embedded liver sections were stained with haematoxylin‐eosin for the histological investigations to evaluate the degree of necrosis after acute liver injury. Additionally, liver sections were stained for Terminal deoxynucleotidyl transferase (TdT) dUTP nick end labeling (TUNEL) assay (Promega) in order to detect apoptotic cells that undergo extensive DNA degradation during the late stages of apoptosis.

### 2.6 Administration of chemical chaperones

Male C57BL/6 (20‐25 g) and their lean controls were purchased from Jackson Laboratories. The control groups used for PBA (4‐phenylbutyrate) treatments received 100 μl PBS twice per day for one week (8 AM and 8 PM) by gavage and the control groups used for TUDCA (taurine‐conjugated ursodeoxycholic acid) treatments received intraperitoneal injection of 100 μl PBS at the same time points (8 AM‐8 PM) for a week. PBA was administered two times a day in two divided doses (250 mg/kg for 8 AM and 8 PM, total 500 mg/kg/day) by oral gavage immediately after the burn injury for one week. TUDCA was applied intraperitoneally at the same time points (250 mg/kg for 8 AM and 8 PM, total 500 mg/kg/day) immediately after the burn injury. Mice tissues were collected post‐treatment and assessed for various measurements described in the manuscript.

### 2.7 Histology and Immunohistochemistry

Liver tissue was immediately fixed in 10% formalin and then maintained in 70% ethanol prior to paraffin embedding. Subsequently, tissues were sectioned and stained with Haematoxylin and Eosin (H&E) or incubated with Ki‐67 (Cell Signaling) antibody followed by DAB staining. Some livers were also immediately frozen in a tissue freezing medium (OCT compound, Tissue‐Tek) and stored at –80°C until staining for Oil Red O. Imaging was performed on a LSM confocal microscope (Zeiss, Germany).

### 2.8 Quantitative PCR

Total RNA isolated from liver tissue was analysed by quantitative RT‐PCR. RNA was isolated from tissue using TRIzol‐chloroform (Life Technologies) with subsequent purification using the RNeasy Kit (Qiagen) according to the manufacturer's instructions. RNA (2 µg) was transcribed to cDNA using the high‐capacity cDNA reverse transcription kit (Applied Biosystems). Real‐time quantitative PCR was performed using the Applied Biosystems Step One Plus Real‐Time PCR System. Primer sequences used are available upon request.

### 2.9 Microarray analysis

The liver was dissected from each mouse and homogenized, total RNA was extracted using a QIAGEN kit according to the manufacturer's instructions. For gene profile analysis, RNA quality was assessed with a Bioanalyzer (Agilent Technologies), and samples with an RNA purity greater than 1.8 were included for array. cDNA was generated and hybridized onto the Affymetrix Mouse Gene 1.0 ST chips. Analysis of gene expression was performed using Parktec Genotyping Suite and Ingenuity Systems Software.

### 2.10 Statistical analysis

All data are presented as mean ± SEM and analysed using Prism (Graphpad). Statistical significance was determined using a Student's t test or one‐way ANOVA followed by a bonferroni post hoc tests as indicated. A *P* value of < 0.05 was considered to be statistically significant, and is presented as * (*P* < 0.05).

## 3 RESULTS

### 3.1 Hepatic response to burn injury

**FIGURE 1:**
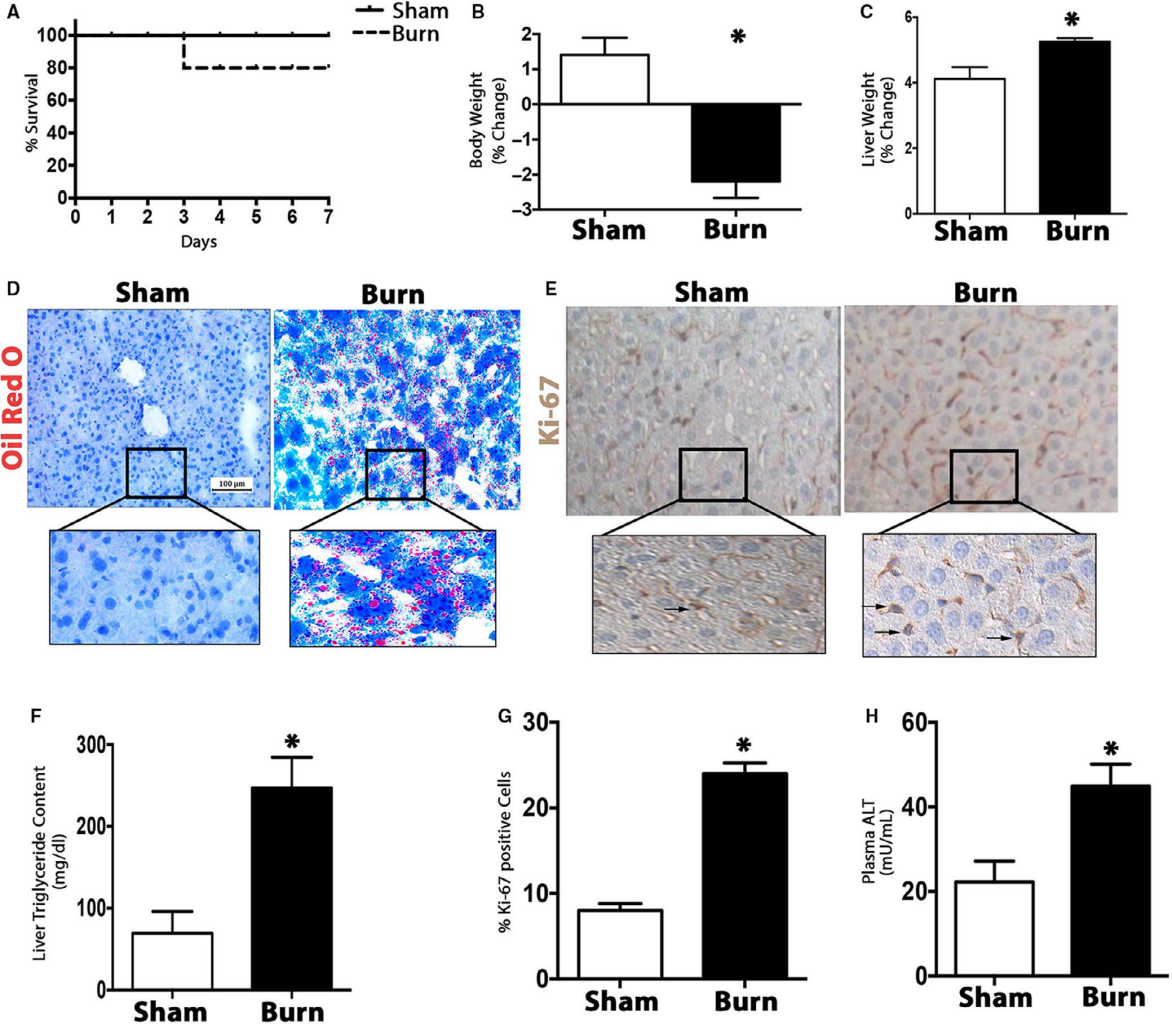
Decreased survival after a burn injury is associated with hepatic dysfunction. A, Kaplan‐Meier survival curve of control mice and mice subjected to a 30% total body surface area thermal injury. B and C, Changes in total body and liver weights in post‐burn and control mice. D, Oil Red O staining for fat droplets in liver sections from burned mice and controls. E, Hepatocyte proliferation detected by immunoperoxidase staining for Ki‐67 in liver sections from burned mice and controls. F, Triglyceride (TG) content of livers from burned mice and controls. G, Quantification of Ki‐67 positive cells in liver sections from burned mice and controls. H, Plasma levels of alanine aminotransferase (ALT) in burned mice and controls. Data represented as mean ± SEM, *P* < 0.05 * = significant difference burn vs. controls (n = 6)

Many severe burn patients with chronic hypermetabolism often succumb to the injury due to the dysfunction of vital organs, such as the liver. Given that hepatic steatosis and dysfunction contribute to poor patient outcomes, we first characterized the hepatic changes that occur in response to hypermetabolism induced by a traumatic injury. Using a mouse model of thermal injury, we show that severe burns increase mortality as a number of fatalities were observed over the course of 7 days. While control mice had 100% survival, mice subjected to a 30% total body surface area burn injury showed 90% survival (Figure 1A). The burn‐induced increase in mortality was accompanied by significant weight loss, as burn mice lost 2% of their total body weight relative to their control counterparts (Figure 1B). At 7 days post‐burn, liver weight was significantly elevated in the burn group relative to their control counterparts (Figure 1C). This could be explained by the increase in hepatic fat infiltration following injury, which we demonstrated and further confirmed with Oil Red O staining for lipid droplets (Figure 1D). Consistent with these findings, increased triglyceride accumulation was observed in the liver of burn mice compared to sham (Figure 1F). Our Ki‐67 staining revealed that burn injury in these mice also increased hepatocyte proliferation (Figure 1E and G). To assess how this altered liver functions after injury, we then measured serum levels of the damage marker, alanine aminotransferase (ALT). As expected, ALT was significantly increased in the livers of burned mice, indicating the activation of hepatic apoptotic and regenerative pathways after severe injury (Figure 1H). Taken together, these results indicate that the post‐burn changes in the liver positively correlate with mortality, suggesting hepatic organ dysfunction is an early risk factor for poor outcomes after major trauma. This prompted us to investigate the mechanisms underlying the adverse hepatic alterations that take place after a burn injury.

### 3.2 Molecular mechanisms associated with hepatic dysfunction

**FIGURE 2:**
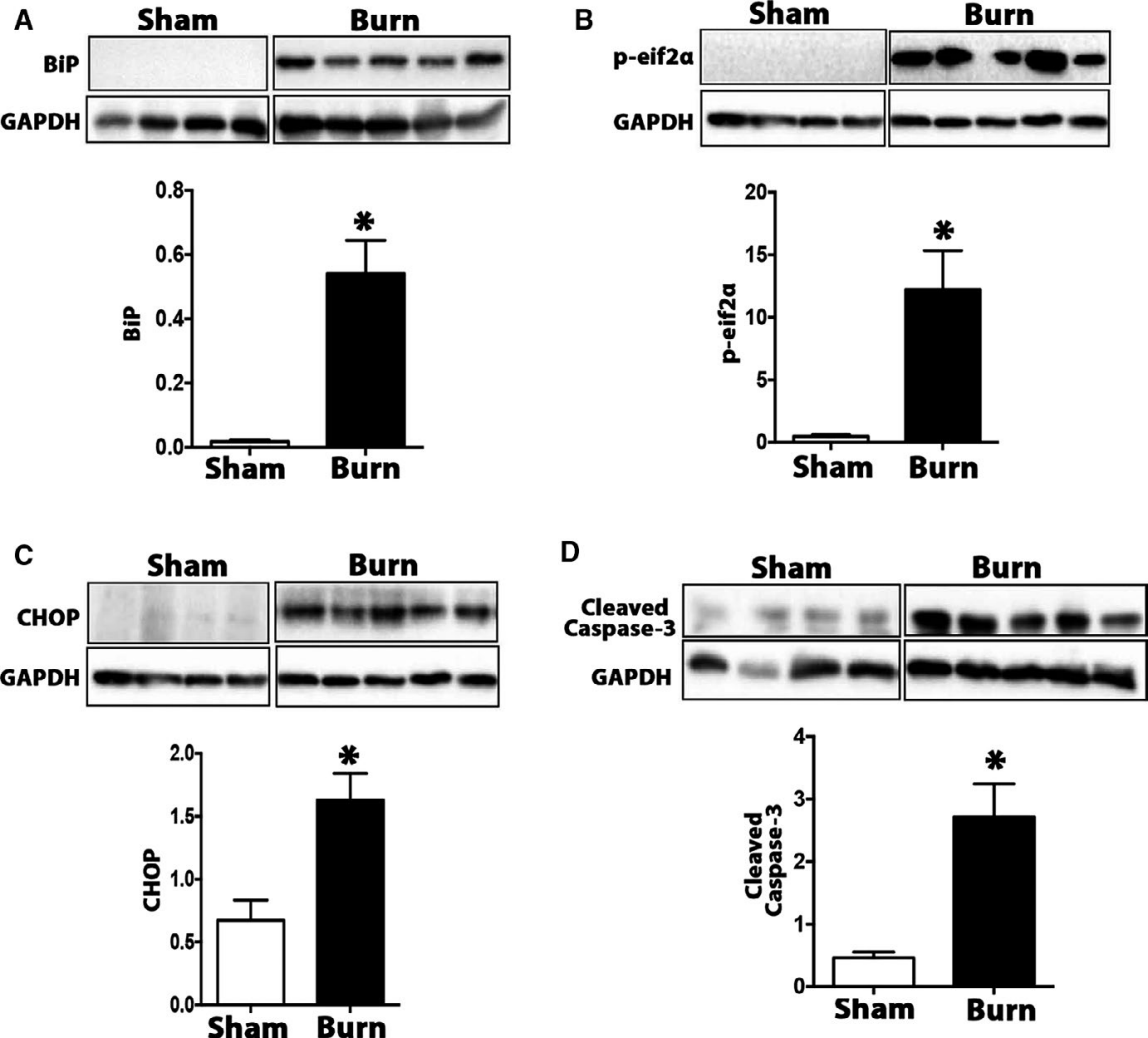
Key ER stress pathways are up‐regulated in the liver after a burn injury. A, Imunoblot and quantification of ER stress marker BiP in livers from burned mice and controls. B, Immunoblot and quantification of ER stress marker p‐eif2a in livers from burned mice and controls. C, Immunoblot and quantification of ER stress marker CHOP in livers from burned mice and controls. D, Immunoblot and quantification of cell apoptosis marker cleaved caspase‐3 in livers from burned mice and controls. GAPDH was used to normalize the immunoblot quantification data and utilized as a loading control. Data represented as mean ± SEM, *P* < 0.05 * = significant difference burn vs. controls (n = 6)

Since numerous studies have indicated a crucial role of ER stress and the UPR signalling pathways in the pathogenesis of liver diseases,^14^, ^20^, ^21^ we decided to assess whether the ER stress response also played a role in the liver following burn injury. Indeed, we observed a robust activation of key ER stress markers in the livers of mice at 7 days post‐burn. As shown in Figure 2A, protein expression of the ER stress‐sensing molecule, BiP or GRP78, was significantly increased in the livers of burn mice compared to control. In addition, burn injury increased the phosphorylation of the downstream ER stress initiation factor eIF2a, thereby increasing its enzymatic activity as a protein translation inhibitor (Figure 2B). Interestingly, the ER stress protein C/EBP homologous protein (CHOP), which is both a transcriptional activator and sensitizer of the intrinsic apoptotic pathway, was also found to be up‐regulated in the livers of burn mice (Figure 2C). These data were further corroborated by the increase in activation of the pro‐apoptotic protein cleaved caspase 3, in the liver post‐burn (Figure 2D). Overall, these findings support the activation of ER stress as a potential mechanism underlying hepatic organ dysfunction after burn. As such, we next addressed whether increased ER stress impacts survival outcomes after a burn injury.

### 3.3 Increased hepatic ER stress leads to burn‐induced mortality

**FIGURE 3:**
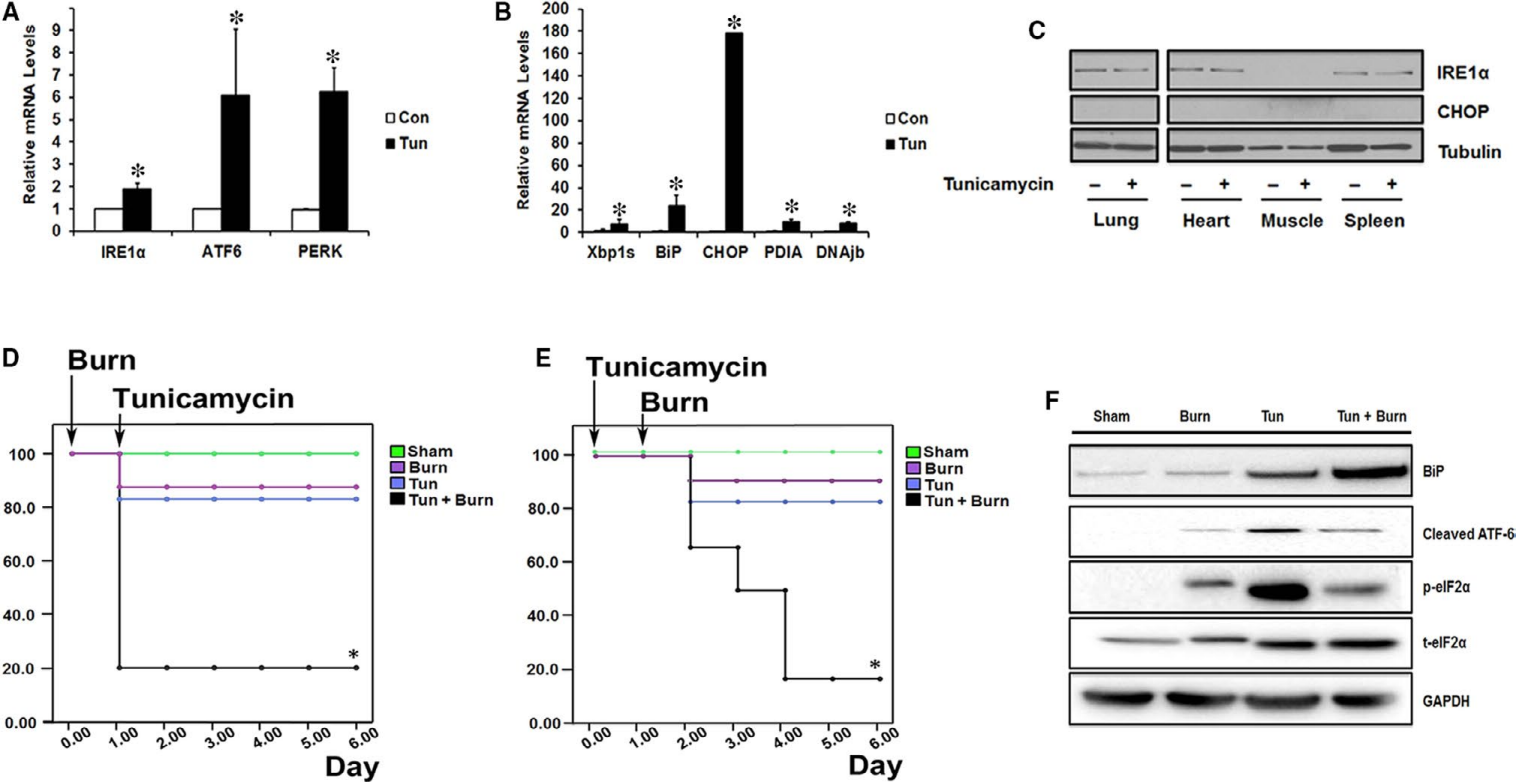
Augmented ER stress following burn injury leads to decreased survival. A and B, Key ER stress/UPR gene markers in livers from tunicamycin‐treated mice and controls. C, Immunoblot of key ER stress markers in lung, heart, muscle and spleen tissue samples taken from tunicamycin‐treated mice and controls. D and E, Kaplan‐Meier survival curves of mice injected with tunicamycin either after (D) or before (E) burn. F Immunoblot of ER stress/UPR proteins in liver samples of Sham, Burn, Tun. and Tun + Burn mice. Tun.; tunicamycin. GAPDH/ or Tubulin respectively was used to normalize the immunoblot quantification data and utilized as a loading control. Data shown are in mean ± SEM, *P < 0.05 vs sham. Tun;Tunicamycin, (n = 6)

Given that morbidity and mortality in 30% TBSA scald burn injury model are low^20^ and that the ER stress response is only moderately increased, we added a ‘second hit’ in an attempt to further augment the ER stress response. To achieve this, we took advantage of the ER stress inducer tunicamycin,^22^ a well‐known inhibitor of protein glycosylation. As illustrated in Figure 3A‐C, administration of tunicamycin by intraperitoneal (IP) injection effectively induced all branches of the ER stress pathway in the liver after treatment in mice. Interestingly, tunicamycin injection alone resulted in a similar number of fatalities as the severe burn injury. While no difference in mortality was observed when administered independently, the combination of tunicamycin and burn injury further increased mortality rates to 80% when compared to mice with burn alone, tunicamycin alone or control. Animals receiving both treatments also presented with a substantial increase in hepatic ER stress, suggesting hyperactivation of the response may be linked to the poor outcomes (Figure 3D). To confirm the detrimental effect of tunicamycin on survival rates, we compared the effects of administering tunicamycin at different time points. Indeed, increased mortality in treated mice was independent of whether tunicamycin was given before or after the burn injury (Figure 3D‐E). To assess whether the substantial increase in mortality was a consequence of ER stress, we analysed the activation of key ER stress pathways and their downstream signalling: IRE1α, PERK, ATF6. As shown in Figure 3F, proteins involved in all the ER stress branches were up‐regulated in the treated mice. These results provide compelling evidence that unmitigated chronic ER stress signalling can adversely influence survival outcomes. Based on these findings, we next assessed the pathophysiological effects of augmented ER stress activation after a burn injury.

### 3.4 Augmented ER stress post‐burn injury mediates hepatic steatosis and dysfunction

**FIGURE 4:**
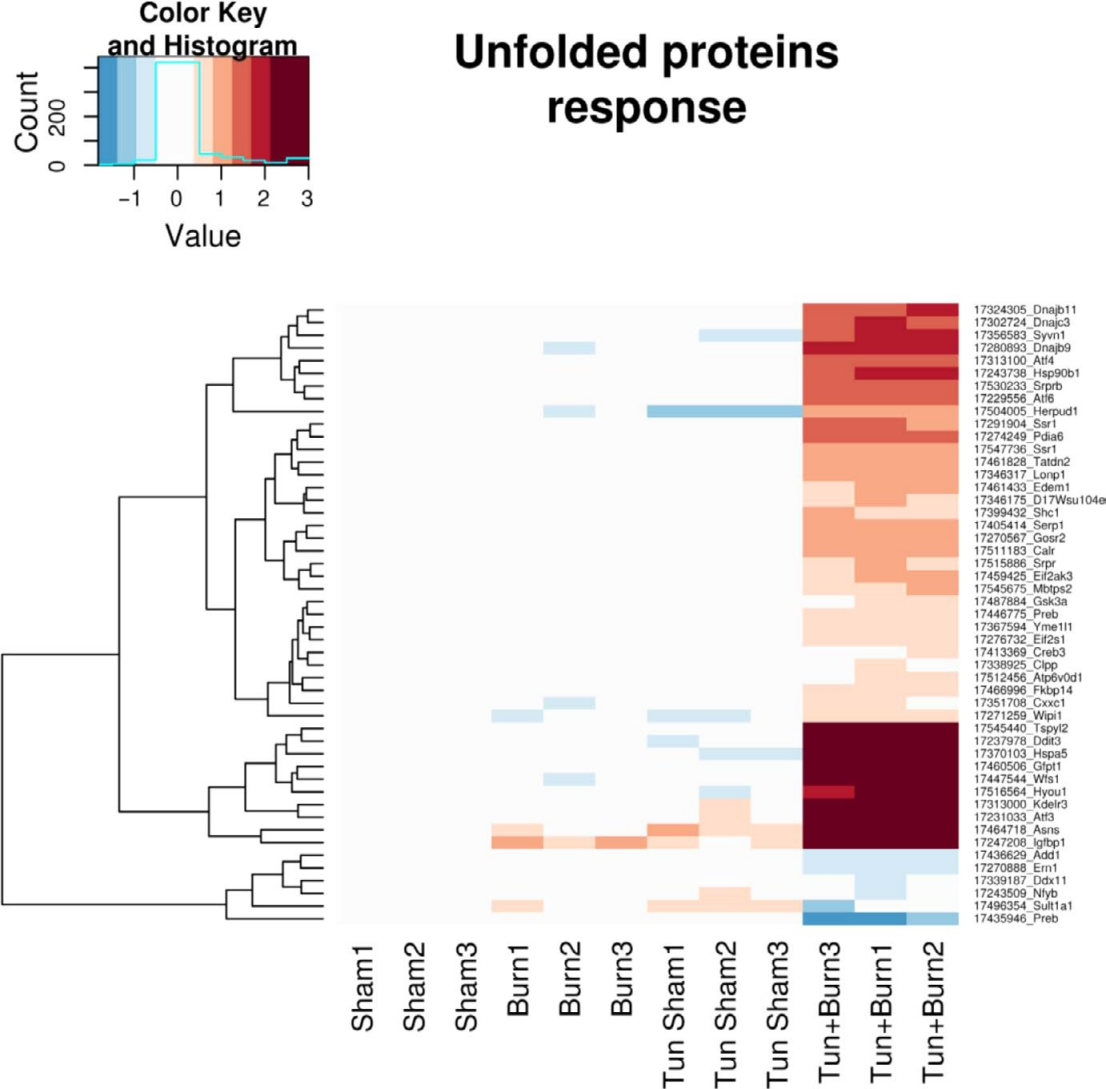
Tunicamycin treatment in post‐burn mice induces alterations in hepatic genes involved in the unfolded protein response and ER stress. Parametric analysis of gene‐set enrichment (PAGE) of the most highly up‐regulated (red) and down‐regulated (blue) unfolded protein response/ER stress genes of livers from controls, burn, tun and tun + burn mice. Tun;Tunicamycin. (n = 3)

**FIGURE 5:**
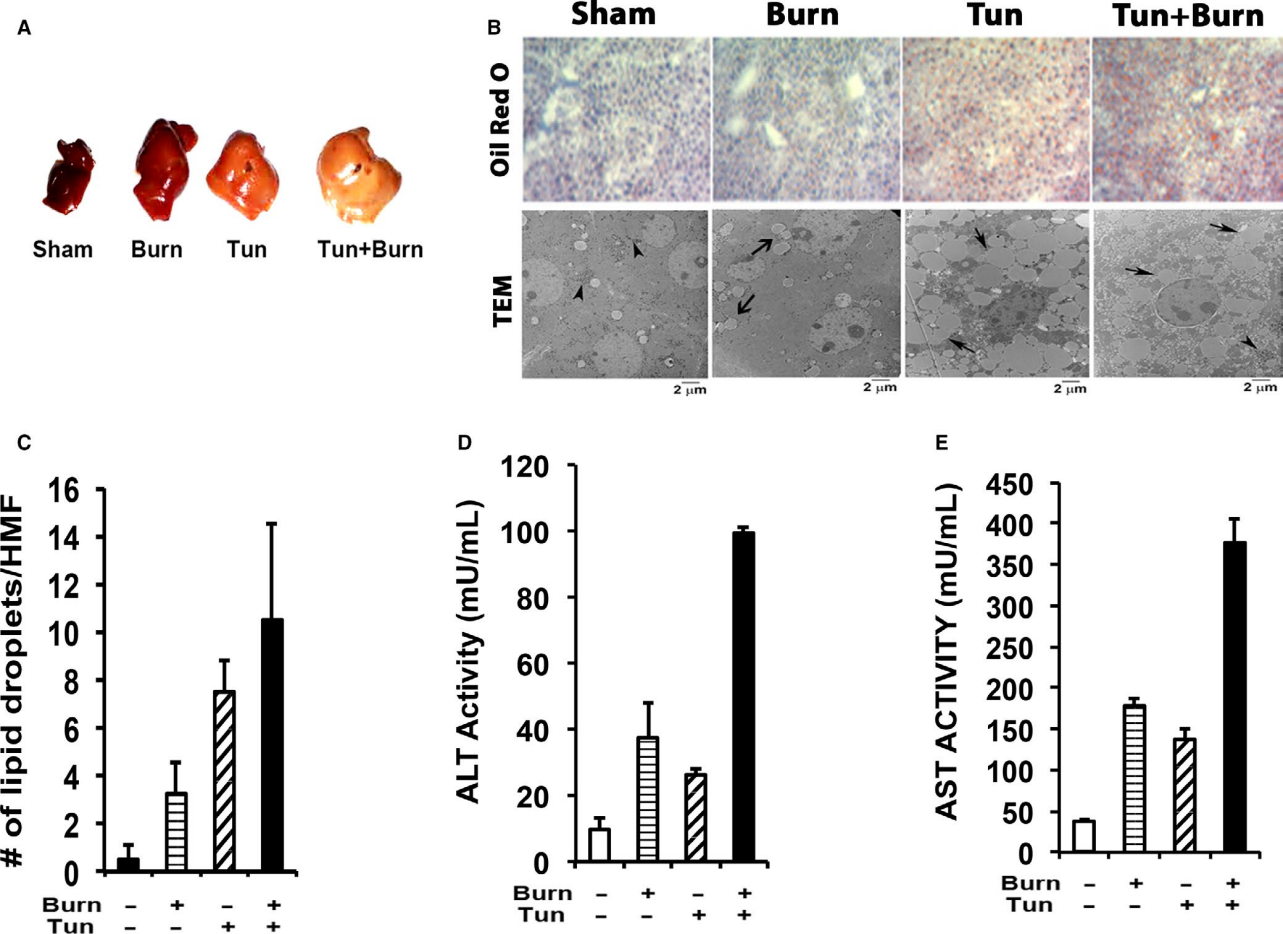
Augmented ER stress following burn injury leads to hepatic steatosis and dysfunction. A, Histopathology of mouse liver tissue from Sham, Burn, Tun. and Tun + Burn post‐injury. B, Oil Red O and transmission electron microscopy staining for lipid droplets in liver sections from Sham, Burn, Tun. and Tun + Burn mice. C, Quantification of the number of lipid droplets in liver sections from Sham, Burn, Tun. and Tun + Burn mice. D and E, Plasma levels of alanine aminotransferase (ALT) and aspartate aminotransferase (AST) in Sham, Burn, Tun. and Tun + Burn mice. Tun;Tunicamycin. Scale bar = 20X. Data shown are in mean ± SEM, *P < 0.05 vs sham, #*P* < 0.05 vs burn. (n = 5‐7 in each group)

Given that burn injury triggered activation of the hepatic ER stress response, we performed genomic profiling of livers taken from control, burn, and tunicamycin‐treated mice to further corroborate augmented ER stress activation. As illustrated in Figure 4, mice who received burn injury alone or in combination with tunicamycin demonstrated substantial alterations in genes involved in the unfolded protein response, which has been shown to regulate cellular and lipid metabolism. Gross pathological examination of the livers isolated from both these groups also showed increased hepatomegaly and hepatic steatosis, with greatest severity observed in the burn plus tunicamycin group (Figure 5A). This organ damage was further confirmed via histological examination of the liver tissue sections. Our Oil Red O staining for lipids followed by transmission electron microscopy revealed the accumulation of large lipid droplets in the livers of both burn and burn plus tunicamycin‐treated mice (Figure 5B and C). To assess whether augmented ER stress directly altered liver function, we then compared serum levels of hepatic damage markers between the groups. Serum levels of AST and ALT were significantly increased by both burn and tunicamycin alone. However, the greatest elevation was observed in the burn plus tunicamycin‐treated group indicating significant hepatic dysfunction in these mice (Figure 5D and E). Collectively, our findings suggest that chronic UPR/ER stress activation augments hepatic dysfunction post‐burn, potentially affecting survival outcomes after injury.

### 3.5 Attenuation of ER stress via chemical chaperones post‐burn injury

**FIGURE 6:**
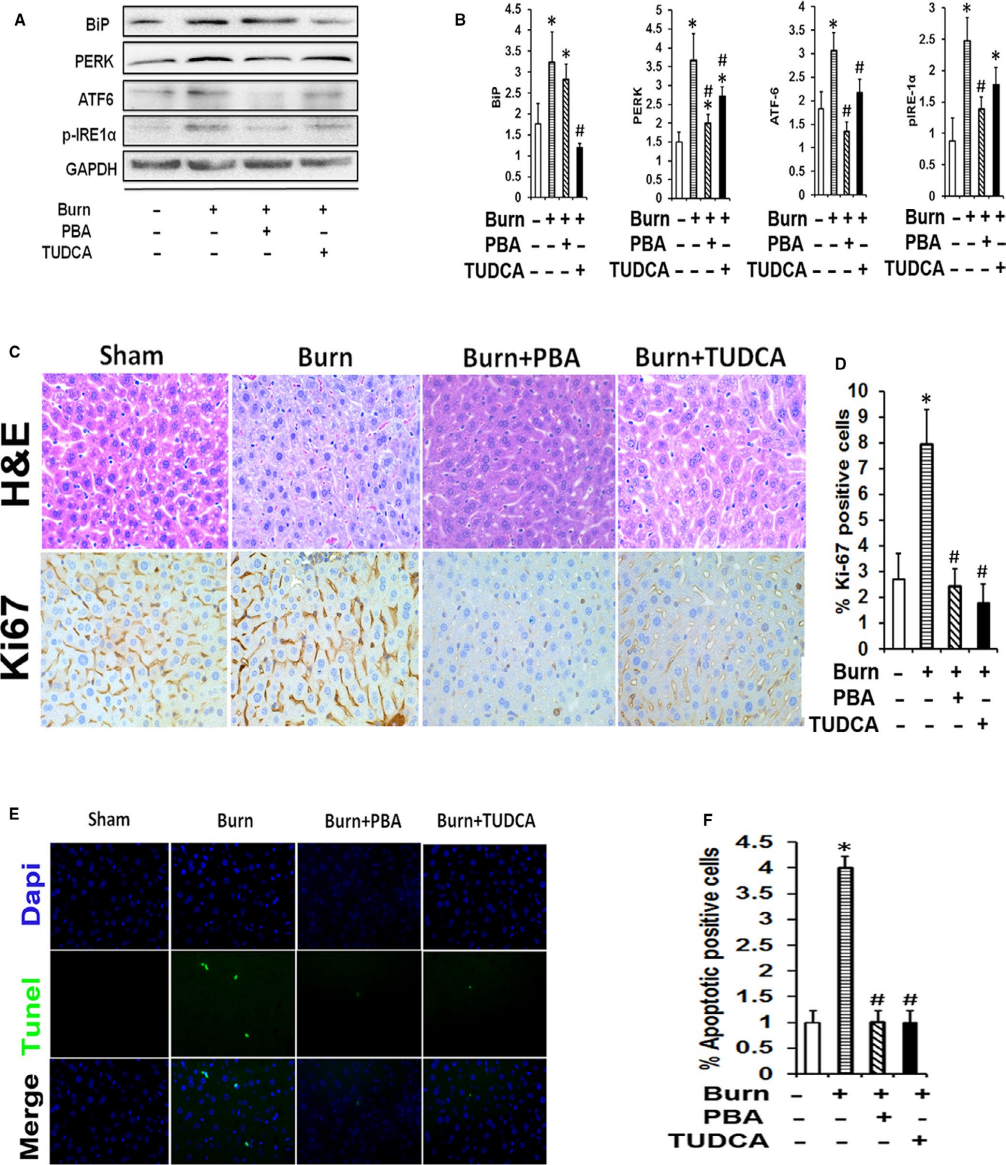
Reduction of burn‐induced hepatic ER stress and apoptosis by chemical chaperones. A and B, Representative Western blot and densitometry quantification of ER stress markers (BiP, PERK, ATF6 and phosphor‐IRE1α) in livers from burn, burn + chaperone treatment (PBA/TUDCA) and control mice. C, Haematoxylin and Eosin (H&E) and immunoperoxidase staining for Ki‐67 in livers from burn, burn + chaperone treatment (PBA/TUDCA) and control mice. D, Quantification of Ki‐67 positive cells in livers from burn, burn + chaperone treatment (PBA/TUDCA), and control mice. E, Fluorescent terminal transferase‐mediated dUTP‐biotin nick end labeling (TUNEL) assay for apoptosis (green) and nuclei (blue) counterstained with 4,6‐dimadino‐2‐phenylindoyl (DAPI) in livers sections from burn, burn + chaperone treatment (PBA/TUDCA) and control mice. F, Quantitative analysis of the number of apoptotic cells in livers sections from burn, burn + chaperone treatment (PBA/TUDCA), and control mice. Scale bar = 20X. GAPDH was used to normalize the immunoblot quantification data and utilized as a loading control. Data shown are in mean ± SEM, *P < 0.05 vs sham, #*P* < 0.05 vs burn. (n = 5‐7 in each group)

Having established a clear interconnection between ER stress, mortality and morbidity, we subsequently investigated whether alleviating ER stress could improve post‐burn survival outcomes. To test this, we took advantage of two FDA‐approved treatments known to alleviate ER stress in conditions outside of burns, TUDCA and PBA.^23^ Using these chemical chaperones, we examined the potential therapeutic effects of inhibiting post‐burn hepatic stress in our hypermetabolic trauma model. To accomplish this, mice were administered a 30% TBSA burn and were randomized to vehicle, burn, burn plus TUDCA (500 mg/kg/day) or burn plus PBA (500 mg/kg/day). While burn injury induced hepatic ER stress reflected by activation of BiP, ATF‐6, PERK, IRE‐1, treatment with TUDCA or PBA attenuated the expression of these ER stress proteins in post‐burn mice (Figure 6A and B). Reduced hepatic ER stress was associated with improved liver morphology (reduced lobular necroinflammation and fibrosis), reduced cell death and subsequent proliferation (Figure 6C‐F). Thus, these findings implicate hepatic ER stress as a detrimental factor during a burn injury, and that the deleterious effects of ER stress can be improved with the use of agents that target ER stress directly.

## 4 DISCUSSION

Current understanding of the activation of ER stress during hypermetabolic conditions is poorly understood, and whether such ER stress activation has deleterious effects on outcome and organ function is even less known. In this study, we provide several lines of evidence to support the detrimental adverse effects of ER stress activation during periods of hypermetabolism that is burn trauma. Firstly, we show that increased ER stress activation during a hypermetabolic state (burns) increases mortality. Secondly, our data implicate organ failure as the mediator of poor outcome under increased ER stress conditions. Finally, the inhibition of ER stress activity in a hypermetabolic condition (burns) through the use of chemical chaperones significantly attenuates hepatic ER stress and improves organ function.

The striking mortality in the burned mice with augmented ER stress uncovers a detrimental aspect to the hyperactivation of the ER stress cascade than its previously known beneficial function in improving and restoring cellular homeostasis. Our observations also implicate the liver as a central determinant in post‐burn survival. In a recent study, Price and colleagues have assessed the outcomes of 290 burned patients who suffered from liver disease prior to burn injury.^24^, ^25^ They found that pre‐existing liver disease increased mortality risk from 6% (total population) to 27%. In fact, this increased risk held when they matched for propensity score and well as for demographics and medical comorbidities.^24^, ^25^ The authors concluded that liver impairment worsens prognosis in patients with thermal injury and that liver integrity is critical for post‐burn survival.^24^, ^25^ Our data confirm these findings and unravel a mechanism by which a burn injury mediates its adverse effects on the liver, helping to shed some light on a very complex aspect of severe burns.

While the exact mechanisms of how ER stress fosters organ failure remain unclear, it likely involves the mitochondria. In fact, ER‐mitochondria cross talk has been shown to be crucial for metabolic homoeostasis and the regulation of cell death.^26^ Several reports have also indicated that depletion of the proteins involved in the regulation of mitochondrial‐ER cross talk, such as mTOR, leads to increased apoptosis, autophagy and cellular dysfunction.^27^, ^28^, ^29^ Conversely, it has also been shown that artificially increasing ER‐mitochondria contacts in cells restore cell viability.^30^ These findings highlight the complex nature of the ER‐mitochondria interface, as either increasing or decreasing cross talk between these two organelles can beneficial or deleterious. Thus, one of the limitations of our study is that we were unable to determine whether the observed hepatic ER stress also had effects on mitochondrial function and proteins that regulate ER‐mitochondria cross talk. As such, further studies are warranted to tease out how hepatic ER stress influences cell death and survival pathways in conditions of trauma.

The last aspect of our study was geared towards clinical applications. We tested the hypothesis that reduction of the increased ER stress response during hypermetabolic states is beneficial in terms of organ function. Treatment of hypermetabolic mice with TUDCA or PBA resulted in diminished hepatic ER stress and organ function, as well as the mitigation of intracellular signalling pathways involved in cell death. Previous reports indicated a role and function for chaperones in diabetes,^23^ while this study is the first to show a potential benefit of chemical chaperones in a hypermetabolic traumatic injury.

Some limitations of our study deserve further discussion. For example, we only used pharmacological inhibitors without tissue or cell type‐specific genetic inhibitory approaches to fully illustrate that the ER inhibitory effects of the chemical chaperones we used were specific to reducing ER stress, rather than mitigating a systemic factor like cytokines that have been shown to activate ER stress. We also did not study further the effects of the chaperones on other organs like the adipose tissue that have been shown to secrete free fatty acids to exert its ER stress effects on the liver. Finally, it is also important for future studies to study longer time points than the 1‐week time point utilized in our study, as this will help to characterize the long‐term safety profile of using the two chemical chaperones in burn trauma.

Our finding that burn causes ER stress and UPR activation presents a pivotal new direction in burn research, facilitating the development of novel therapeutic strategies and improved clinical outcomes for those with severe burn injuries. Specifically, our findings have identified that intact liver function is critical for burn trauma outcomes, and therapeutic interventions that reduce factors that promote hepatic dysfunction could be worthwhile in reducing the morbidity of burn patients. Indeed, our novel findings using the chemical chaperones, PBA and TUDCA, which have outstanding in vivo safety profiles, may be a useful therapeutic strategy in this regard. The clinical translational potential of both PBA and TUDCA use burn patients is also promising since both have already been approved by the US Food and Drug Administration for clinical use in a number of conditions like thalassaemia, cystic fibrosis, cholestatic liver diseases.^31^ Future studies should commence a small safety profile clinical trial of PBA and TUDCA use in burn patients.

In conclusion, our in vivo findings indicate that augmented ER stress can impact survival, additionally addressing the essential role of this response in determining outcome after a burn trauma. Our work also offers tools to modulate the ER stress response associated with hypermetabolic conditions (burns, cancer and heart disease) and illustrates the therapeutic effects in potentially improving outcomes for patients.

## 5 CONFLICTS OF INTEREST AND SOURCE OF FUNDING

This study was supported by National Institutes of Health R01‐GM087285‐01. CFI Leader's Opportunity Fund: Project #25407 and Canadian Institutes of Health Research (CIHR) grant #123336. Authors have no other conflicts of interest to declare.

## AUTHOR CONTRIBUTION

Abdikarim Abdullahi: Conceptualization (equal); Data curation (lead); Formal analysis (lead); Writing‐original draft (lead). Dalia Baryan: Data curation (supporting); Formal analysis (supporting); Writing‐original draft (supporting). Roohi Vinaik: Data curation (supporting); Formal analysis (supporting); Writing‐original draft (supporting). Li Diao: Data curation (supporting); Formal analysis (supporting). Nancy Yu: Data curation (supporting); Formal analysis (supporting); Writing‐original draft (supporting). Marc G Jeschke: Conceptualization (lead); Formal analysis (supporting); Funding acquisition (lead); Writing‐original draft (supporting).

## Data Availability

The data that support the findings of this study are available from the corresponding author upon reasonable request.
